# A Treatise on the Nervous Diseases of Children, for Physicians and Students

**Published:** 1895-10

**Authors:** 


A Treatise on the Nervous Diseases of Children, for Physicians and Students. By B. Sachs, M. D., Professor of Mental and Nervous Diseases in the New York Polyclinic; Consulting Neurologist to the Mt. Sinai Hospital; Neurologist to the Montefiore Home for Chronic Invalids; ex-President of the American Neurological Association. One volume, pp. 688, 8vo, illustrated by 169 engravings in black and color and a colored plate. Muslin, $5.00. New York: William Wood & Company. 1895.

Perhaps no one is more better fitted to write a treatise on the nervous diseases of children than Professor Sachs, and the manner in which he has accomplished the task is proof of his ability in this direction. Just why the title of the book should be restricted to the nervous disturbances of childhood, when, in fact, the large majority of nervous diseases affect both young and old alike is questionable, and it seems to narrow the field of its distribution and consequently accessibility.

We cannot quite agree that its limitation to the diseases of children is a peculiarly happy one, inasmuch as it is not monographic enough for special workers and yet too full and complete for the average busy practitioner or time-pressed medical student. It does serve, however, to place the author's views and beliefs on record and adds another volume to the excellent list of American neurologies. The reviewer believes that every original worker or thinker should leave some record or archive in which his views can be easily consulted and is extremely glad that Professor Sachs has in this volume found expression for his wide experience, judicious selection of facts and personal investigations. The stamp of a master-mind is imprinted on every page and the book, consequently, bristles with knowledge of the greatest worth to workers in the field of neurology.

Instead of beginning with the organic diseases, the author starts out with a description of the functional diseases, convulsions, epilepsy, hysteria, chorea and the like. The surgical treatment of the eye muscles for the cure of epilepsy is utterly condemned and the operation of trepanation is not urged with as much fervor as was done a few years ago. As regards hysteria, the author states that it is a relatively rare disease in the adult and still rarer in the child; and this has been the experience of the reviewer, although to the general practitioner it seems to be an ever-present malady. He further states that the Anglo-Saxon race is less prone to the development of hysteria than the other races represented in our population. These chapters are well presented, the facts well brought out and clearly elucidated.

Somewhat disappointing is the short article on enuresis nocturna, many forms of which are of nervous origin and not due to indifference, laziness or wilfulness. This annoying condition is extremely often met with by the general practitioner and specialist in neurology and should receive, somewhere, a very careful analysis and investigation, and where better than in a treatise on the nervous diseases of children?

On the organic diseases of the cord and brain, muscular atrophies and the like, the author is at his best and a large experience enables him to present these chapters in a masterful manner. The cerebral and spinal palsies of children and different types of muscular atrophy have attracted the author's attention for some years past, and their succinct portrayal at this place is eagerly welcomed by all readers of neurological literature. The closing chapters are devoted to the consideration of mental disturbances of childhood, hardly enough attention being given to idiocy, imbecility and cretinism, affections of childhood par excellence. In regard to partial or defective development of the brain, the author does not believe that Lannelongue's operation is likely to permanently increase the intra-cranial capacity, nor does he consider that a small skull is the primary or even the most important cause of arrested development of the brain. The fault lies chiefly with the brain itself and cannot be remedied by operation. One hundred and sixty-two nicely executed illustrations adorn the work, the majority of which are original and here published for the first time, giving the book a sparkle and brilliancy in keeping with its reading matter. It will, no doubt, take front rank with the very best text-books on neurology in any language.

The publishers have succeeded admirably in doing what was expected from the William Wood Company. W. C. K.

